# Fuzzy Multi-SVR Learning Model for Reliability-Based Design Optimization of Turbine Blades

**DOI:** 10.3390/ma12152341

**Published:** 2019-07-24

**Authors:** Chun-Yi Zhang, Ze Wang, Cheng-Wei Fei, Zhe-Shan Yuan, Jing-Shan Wei, Wen-Zhong Tang

**Affiliations:** 1 School of Mechanical and Power Engineering, Harbin University of Science and Technology, Harbin 150080, China; 2 Department of Aeronautics and Astronautics, Fudan University, Shanghai 200433, China; 3 School of computer Science and Engineering, Beihang Unviersity, Beijing 100191, China

**Keywords:** reliability-based design optimization, turbine blades, multi-objective genetic algorithm, fuzzy support vector machine of regression, uncertainty

## Abstract

The effectiveness of a model is the key factor of influencing the reliability-based design optimization (RBDO) of multi-failure turbine blades in the power system. A machine learning-based RBDO approach, called fuzzy multi-SVR learning method, was proposed by absorbing the strengths of fuzzy theory, support vector machine of regression (SVR), and multi-response surface method. The model of fuzzy multi-SVR learning method was established by adopting artificial bee colony algorithm to optimize the parameters of SVR models and considering the fuzziness of constraints based on fuzzy theory, in respect of the basic thought of multi-response surface method. The RBDO model and procedure with fuzzy multi-SVR learning method were then resolved and designed by multi-objective genetic algorithm. Lastly, the fuzzy RBDO of a turbine blade with multi-failure modes was performed regarding the design parameters of rotor speed, temperature, and aerodynamic pressure, and the design objectives of blade stress, strain, and deformation, and the fuzzy constraints of reliability degree and boundary conditions, as well. It is revealed (1) the stress and deformation of turbine blade are reduced by 92.38 MPa and 0.09838 mm, respectively. (2) The comprehensive reliability degree of the blade was improved by 3.45% from 95.4% to 98.85%. (3) It is verified that the fuzzy multi-SVR learning method is workable for the fuzzy RBDO of complex structures just like a multi-failure blade with high modeling precision, as well as high optimization, efficiency, and accuracy. The efforts of this study open a new research way, i.e., machine learning-based RBDO, for the RBDO of multi-failure structures, which expands the application of machine learning methods, and enriches the mechanical reliability design method and theory as well.

## 1. Introduction

The reliability-based design optimization (RBDO) of mechanical structures is usually adopted to find the optimal structural design parameters such as mass, structural sizes, and so forth, by establishing the RBDO model, subject to the relevant parameters (i.e., material properties, complex loads), comprehensive reliability index, and the technical and economic requirements of structural design [1,2]. The RBDO of complex structures commonly involves many failure modes and holds the features of nonlinearity, fuzziness, and randomness. For instance, the failure design analysis of the turbine blade involves stress, strain, and deformation affected by the fuzziness and randomness of workloads (temperature, rotor speed, aerodynamic pressure, etc.), material parameters (density, elastic modulus, thermal conductivity, etc.), and constraints [3]. The RBDO of the structures thus has large-scale computational load and complexity and is hardly performed with acceptable efficiency and accuracy by the existing finite element (FE) simulation and stochastic optimization method. To make the optimization design results meet the engineering requirements, it is urgent to develop an effective approach for the RBDO of multi-failure structures.

With the requirement of the design analysis for advanced mechanical systems, numerous RBDO methods have emerged in engineering practice [4,5,6,7]. Response surface method (RSM) is an efficient approach to ensure efficiency and accuracy in structural reliability optimization, instead of the direct simulation method with the FE model [4]. Fei et al. studied extremum response surface methods with quadratic polynomials and support vector regression, for dynamic reliability optimization design of the turbine blade-tip radial running clearance [8,9] and turbine components [10,11]. Song et al. [12] discussed multiple response surface models with a back propagation-artificial neural network, for multi-objective RBDO of turbine blisk, considering fluid-thermal-structure interaction. Chang et al. [13] investigated the constraint-feasible moving-least-squares method for the RBDO of floating production storage and offloading river support structures, regarding operational status, damage, and loading conditions. Youn et al. [14] enhanced RSM with the moving-least-squares method and the hybrid mean-value method, to investigate the RBDO of the vehicle side and improve its crashworthiness. However, the above works only focus on the RBDO problem of structures with single-objective and single-failure mode, rather than the correlation among failure modes and the fuzziness of constraint boundary condition. Hence, it is difficult for these methods to handle the RBDO problems of multi-failure structures with acceptable accuracy and efficiency.

Recently, the machine learning method has had a rapid development in other research fields, such as data-derived modeling and image processing [15,16,17]. The heuristic method leads authors to attempt to expand this idea to engineering optimization. Support vector machine of regression (SVR) is one machine learning method developed from statistical learning theory [10] and has an excellent learning ability of small samples and high-efficient simulation ability, which were validated in the reliability optimization design [18,19,20,21,22]. Recently, Brabanter, et al. [18] proposed the least-squares support vector machine by transforming the optimization problem of SVR into a quadratic programming problem with a single equation constraint, to accelerate support vector regression solution. However, SVR is susceptible to noise and outliers when training samples contain lots of fuzzy information. Therefore, the fuzzy least-squares support vector machine of regression (FLSSVR) was developed by introducing fuzzy membership into SVR, and has been applied in many engineering fields [23]. Up to now, it is not found for the application of FLSSVR in structural reliability optimization designs yet.

The goal of this paper is to attempt to open a new research direction, i.e., machine learning-based RBDO, for mechanical structures with multi-failure modes or multiple components, in respect of the fuzziness of design parameters and constraints. Concretely, a fuzzy multi-SVR learning method is proposed and modeled by adopting an artificial bee colony (ABC) algorithm, to find the optimal parameters of SVR models. The RBDO of a turbine blade with multi-failure modes is implemented based on the fuzzy multi-SVR learning method with a multi-objective genetic algorithm (MOGA) [24,25], regarding the fuzziness of design parameters and constraints. Besides, the proposed approach is validated by the comparison of methods. 

In what follows, the fuzzy multi-SVR learning method is discussed in Section 2, comprising the basic thought of turbine blade RBDO with the fuzzy multi-SVR learning method, the mathematical model of the fuzzy multi-SVR learning method, and the RBDO model. In Section 3, the RBDO of the turbine blade is completed with the fuzzy multi-SVR learning method. Conclusions of this study are summarized in Section 4.

## 2. Theory and Method

As a machine learning method, the support vector machine was first developed for data mining in classification and regression [26,27,28] and is a kind of statistical learning algorithm so that it is promising to be suitable for small samples gained from a handful of FE simulations for structural design analysis. The SVR model does well in solving high nonlinear problems between input parameters and output response, by introducing a maximum classification margin which is actually a quadratic programming problem subjected to inequality constraints [26]. Hence, the SVR has the potential to improve the computational efficiency and accuracy of multi-model structural RBDO [11]. The multi-response surface method (MRSM) was proposed to handle the multi-model problem in the probabilistic analysis of multi-component structures, multi-discipline, and multi-failure modes by assimilating RSM [29,30,31,32]. In most of the structural probabilistic designs, in fact, influential parameters and constraint conditions hold obvious fuzziness and seriously influence design precision. Therefore, it is reasonable to consider the fuzziness of design parameters and constraint conditions to improve the probabilistic design for structures, especially, for multi-failure modes or multi-component structures. In respect of the heuristic thought of MRSM and SVR, this paper develops a fuzzy multi-SVR learning method, a machine learning approach, for the fuzzy RBDO of a multi-failure turbine blade by considering fuzzy parameters and constraints.

### 2.1. Fuzzy Multi-SVR Learning Method

#### 2.1.1. Fuzzy Multi-SVR Learning Model

Assuming that *j*th input sample set of a component in structure system is denoted by ***X***^(*j*)^, and the corresponding output response is *y*^(*j*)^(***X***^(*j*)^), the response surface curve was constructed by the sample set{*y*^(*j*)^(***X***^(*j*)^): *j*∈Z*^+^*}. The relationship between ***X***^(*j*)^ and *y* is expressed by:

(1) $$y=f\left(X\right)=\left\{y^{\left(j\right)}\left(X^{\left(j\right)}\right):j\in Z_{+}\right\}$$

Through multiple fuzzy SVR modeling [26] for *m* failure modes based on multiple response surface method [29], the mathematical model of fuzzy multi-SVR learning method in respect of *m* failure modes is:

(2) $$\left\{ \begin{array}{l} {\tilde{y}}^{\left(1\right)}=f^{\left(1\right)}\left(x\right)=\sum_{i=1}^{l}a_{i}^{\left(1\right)}k^{\left(1\right)}\left(x,x_{i}\right)+b^{\left(1\right)}\\ \begin{array}{cc} & \begin{array}{cc} \vdots & \end{array} \end{array}\\ {\tilde{y}}^{\left(j\right)}=f^{\left(j\right)}\left(x\right)=\sum_{i=1}^{l}a_{i}^{\left(j\right)}k^{\left(j\right)}\left(x,x_{i}\right)+b^{\left(j\right)}\\ \begin{array}{cc} & \begin{array}{cc} \vdots & \end{array} \end{array}\\ {\tilde{y}}^{\left(m\right)}=f^{\left(m\right)}\left(x\right)=\sum_{i=1}^{l}a_{}^{\left(m\right)}k^{\left(m\right)}\left(x,x_{i}\right)+b^{\left(m\right)} \end{array}\right.$$

where *l* is number of support vectors in sample set, *m* denotes number of failure modes; ${\tilde{y}}^{\left(j\right)}$ is the output response with *j*th failure mode (*j =* 1,2,…,*m*), $a_{i}^{\left(j\right)}$ is weight vector with *j*th failure mode, $b^{\left(j\right)}$ is a bias term with *j*th failure mode, $k^{\left(j\right)}(x,x_{i})$ is Kernel function of *j*th failure mode and is expressed with Gauss function as:

(3) $$k^{\left(j\right)}\left(x,x_{i}\right)=\exp\left(-\frac{{\left\|x_{ji}-x_{j}^{'}\right\|}^{2}}{2{\left(\sigma^{\left(j\right)}\right)}^{2}}\right)$$

in which *x_ji_* is *i*th sample in *j*th SVR model, *x_j_^`^* is the center point of sample set ***x****_j_* of *j*th SVR model, *σ* is the width of Gauss Kernel function ***k***^(*j*)^. 

Let $\theta^{\left(j\right)}=\left[a_{i}^{\left(j\right)},b_{i}^{\left(j\right)},\sigma^{\left(j\right)}\right]$ be the parameters of *j*th SVR model ${\tilde{y}}^{\left(j\right)}$ and directly determines the effectiveness of SVR model. Therefore, the parameters ***θ*** should be optimized to improve the modeling precision of SVR model in Equation (2). Search method is the traditional methods in seeking for the parameters ***θ*** and has some blindness as it largely depends on the experience of researchers. Thus, more efficient algorithm should adopt to find the parameters ***θ*** in SVR modeling.

#### 2.1.2. Multi-Objective Genetic Algorithm

**Figure 1:**
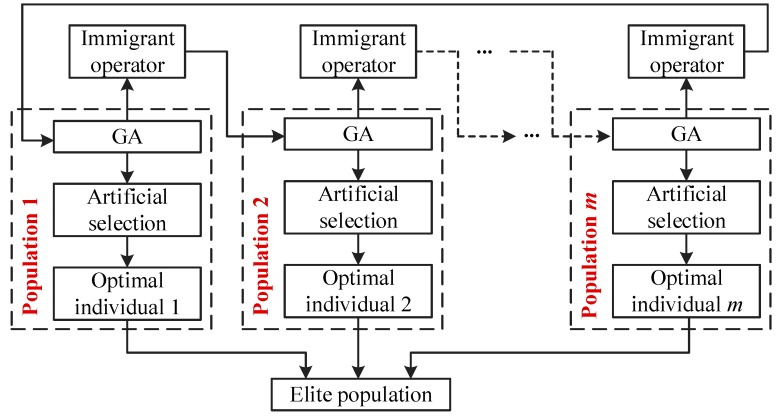
The schematic diagram of multi-objective genetic algorithm (MOGA).

In [33], we investigated the application of the genetic algorithm (GA) in searching the optimal values of Kriging hyperparameter and demonstrated that GA has better advantages than gradient descent optimizer, owing to strong robustness and global searchability. In this paper, the multi-objective genetic algorithm (MOGA) is developed based on the MRSM thought, to find the optimal values of SVR hyperparameter ***θ***. Comparing with GA, MOGA holds more flexible and adaptive design space exploration, and potentially avoid the influence of the plateau-like function profile [34]. Besides, MOGA breaks the limitation of a single population evolution of GA and uses multiple populations with different control parameters for optimization iterations [24,25]. Substantially, MOGA originates from GA and inherits natural selection and genetic characteristics, and the optimal solution of the objective function can be gained via successive iterations with selection, crossover, and mutation. The basic principle of the MOGA is summarized below. We first generate *N* initial populations (i.e., blade density, rotor speed, temperature, aerodynamic pressure, and gravity) with binary encoding, then the *N* new populations are obtained by the procedures of selection operator, crossover operator, and mutation. We further select the optimal individuals of each excellent population via the artificial selection operator, which are applied to the structure elite population to search for the optimal value of the objective function. Obviously, MOGA is essentially a combination of multiple GAs by a certain relationship. However, in MOGA, different control parameters (i.e., crossover probability *p_c_*_,*l*_ and mutation probability *p_m_*_,*l*_) are used to complete the collaborative evaluation of multiple populations (*l* = 1, 2 …, *N*). Thus, the MOGA has both global and local search abilities by introducing immigrant operator to exchange messages among populations and avoid the destruction and loss of optimal individual information. In this process, the elite population does not participate as selection, crossover, or mutation operators. The minimum reserved generation is usually regarded as the terminal condition of optimization iterations. The procedure of MOGA is shown in Figure 1.

### 2.2. RBDO Model with Fuzzy Multi-SVR Learning Method

In this RBDO, highly sensitive input random variables are regarded as design variables, and overall stress and deformation of the structure are considered as design objectives, and the overall reliability *R* of the blade, reliability *R_j_* of *j*th failure mode, and performance constraints allowable value [*σ_io_*] are considered as constraints. In this case, the RBDO model of multi-failure structures with fuzzy multi-SVR learning method is expressed by:

(4) $$\begin{array}{l} \mathrm{Find}\begin{array}{cc} & \underset{˜}{x}= \end{array}{\left({\underset{˜}{x}}_{1},{\underset{˜}{x}}_{2},\cdots ,{\underset{˜}{x}}_{n}\right)}^{T}\\ \min\begin{array}{cc} & \underset{˜}{f} \end{array}\left(x\right)={\left[{\underset{˜}{f}}_{1}(x),{\underset{˜}{f}}_{2}(x),\cdots ,{\underset{˜}{f}}_{i}(x)\right]}^{T}\\ \text{s.t.}\;\left\{ \begin{array}{l} R_{j}\underset{˜}{\geq }\left[R_{j0}\right]\\ R\underset{˜}{\geq }\left[R_{0}\right]\\ {\underset{˜}{f}}_{i}\left(x\right)\underset{˜}{\leq }\left[\sigma_{i0}\right]\\ {\underline{x}}_{i}\underset{˜}{\leq }x_{i}\underset{˜}{\leq }{\bar{x}}_{i}\\ {\underline{x}}_{i}={\underline{x}}_{i}^{l}+\lambda^{*}\left({\underline{x}}_{i}{}^{u}-{\underline{x}}_{i}{}^{l}\right)\\ {\bar{x}}_{i}={\bar{x}}_{i}{}^{u}-\lambda^{*}\left({\bar{x}}_{i}{}^{u}-{\bar{x}}_{i}{}^{l}\right) \end{array}\right. \end{array}$$

where *R_j_* is the reliability degree under *j*th failure mode, [*R_j0_*] denotes the allowable reliability under *j*th failure mode, *R* stands for the comprehensive reliability of multi-failure modes, [*R_0_*] the allowable comprehensive reliability of multi-failure modes, ${\underline{x}}_{i},{\bar{x}}_{i}$ indicate upper and bottom boundary of *i*th fuzzy variables. The upper and bottom bounds of the transitional interval are determined by introducing amplification coefficient, i.e., ${\underline{x}}^{n}=\underline{x}$ ${\underline{x}}^{l}=\underline{\beta }\times \underline{x}$; ${\bar{x}}^{u}=\bar{\beta }\times \bar{x},\;{\bar{x}}^{l}=\bar{x}$; $\left\{\sigma \right\}=\left[\sigma_{x},\sigma_{y},\sigma_{z},\tau_{xy},\tau_{yz},\tau_{zx}\right]$, $\bar{\beta }=1.05\sim 1.3$; $\lambda^{*}$ is the optimal level Scut set.

### 2.3. Flowchart of RBDO with Fuzzy Multi-SVR Learning Method

Based on the proposed multi-SVR learning method, the RBDO procedure of multi-failure turbine blade is described as follows.

***Step 1***: Build the FE model of turbine blades, and select blade density, rotor speed, temperature, aerodynamic pressure, and gravity as input variables, and regard the deformation and stress of turbine blades as two failure modes (i.e., optimized objects), and consider the fuzziness of rotor speed, gas temperature, and boundary conditions. 

***Step 2***: Perform the static deterministic analysis of blades based on FE model and thermal-structural interaction, by regarding the means of random variables, to find the maximum points of blade stress and deformation as the object of turbine blade optimization. 

***Step 3***: Regard the randomness and fuzziness of design parameters to extract handful samples of random variables by Latin hypercube sampling (LHS) technique which have been validated to be a highly effective sampling approach [35], and to calculate the output response of stress and deformation as output samples by FE simulations with the extracted samples of input variables.

***Step 4***: Normalize the samples comprising of input samples and output samples as the training samples to find the optimal parameters of SVR model by using an artificial bee colony (ABC) algorithm, which was verified to be an efficient parameter optimization approach [25], and then to build fuzzy multi-SVR learning models for the deformation and stress of turbine blades.

***Step 5***: Acquire enough samples gained by the linkage LHS technique [35], to conduct the probabilistic simulations of multi-failure structure based on the developed fuzzy multi-SVR learning models.

***Step 6***: Establish the RBDO model with the developed fuzzy multi-SVR learning method by employing the MOGA to find the optimal parameters in the RBDO, in which we regard load parameters (i.e., rotor speed, gas temperature, aerodynamic pressure, etc.) and material parameters as design variables, and stress and deformation as the objective functions (design objectives), as well as reliability and fuzzy boundary conditions as constraint functions.

***Step 7***: Implement the RBDO of a multi-failure turbine blade with the fuzzy multi-SVR learning method to search for the optimal design parameters subject to design objectives and constraints.

**Figure 2:**
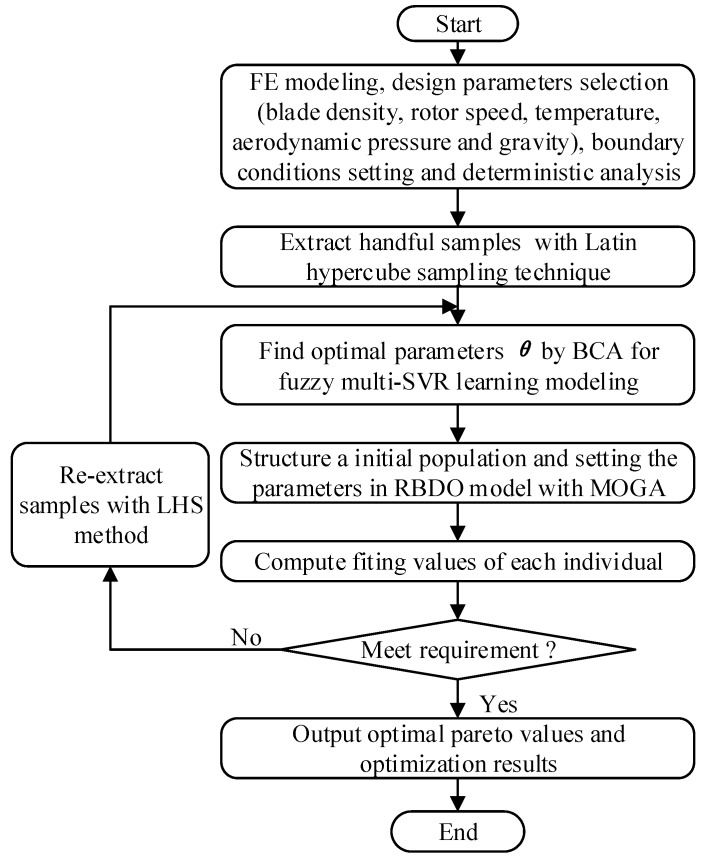
Reliability-based design optimization (RBDO) procedure with fuzzy multi-support vector machine of regression (SVR) learning method.

The above analysis flowchart as summarized in Figure 2.

## 3. Fuzzy Reliability-Based Design of Multi-Failure Turbine Blade

### 3.1. Deterministic Analysis of Turbine Blade

In this paper, the RBDO of an aeroengine turbine blade with multiple failure modes such as stress and deformation were discussed using the fuzzy multi-SVR learning method. The blade material was GH4133B alloy [36]. In respect of the random variable selection method in [37], we considered blade density, rotor speed, gas temperature, aerodynamic pressure, and gravity as input random variables as shown in Table 1. All the variables are assumed to obey normal distributions independently each other.

**Figure 3:**
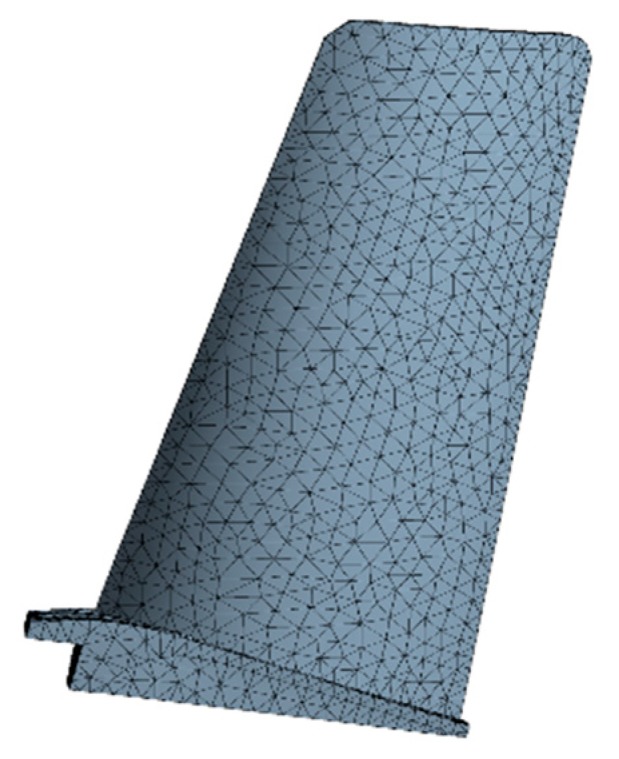
Finite element model of turbine blade.

**Figure 4:**
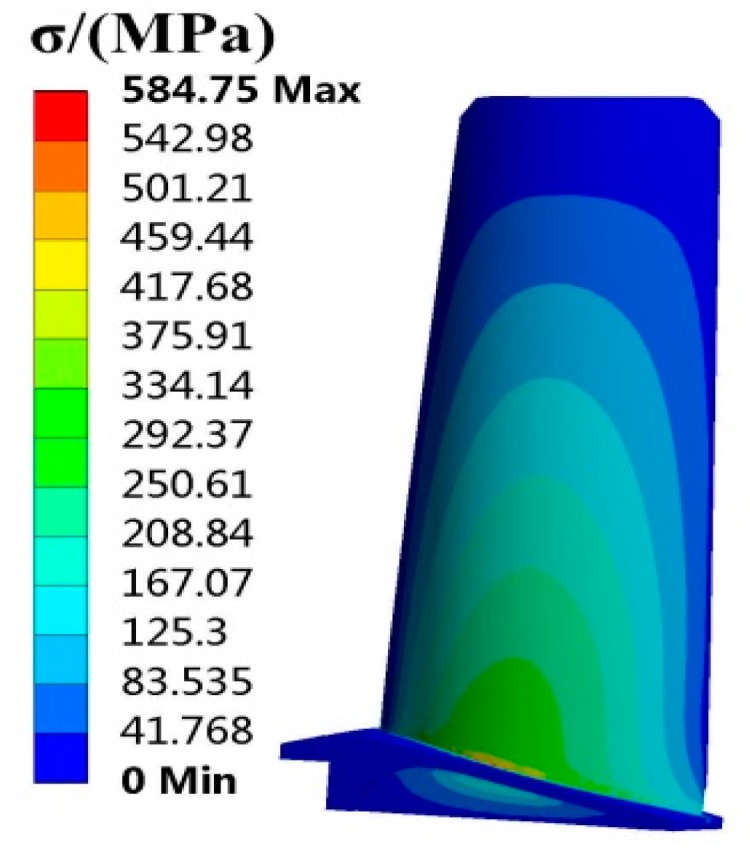
Distribution of blade stress.

**Figure 5:**
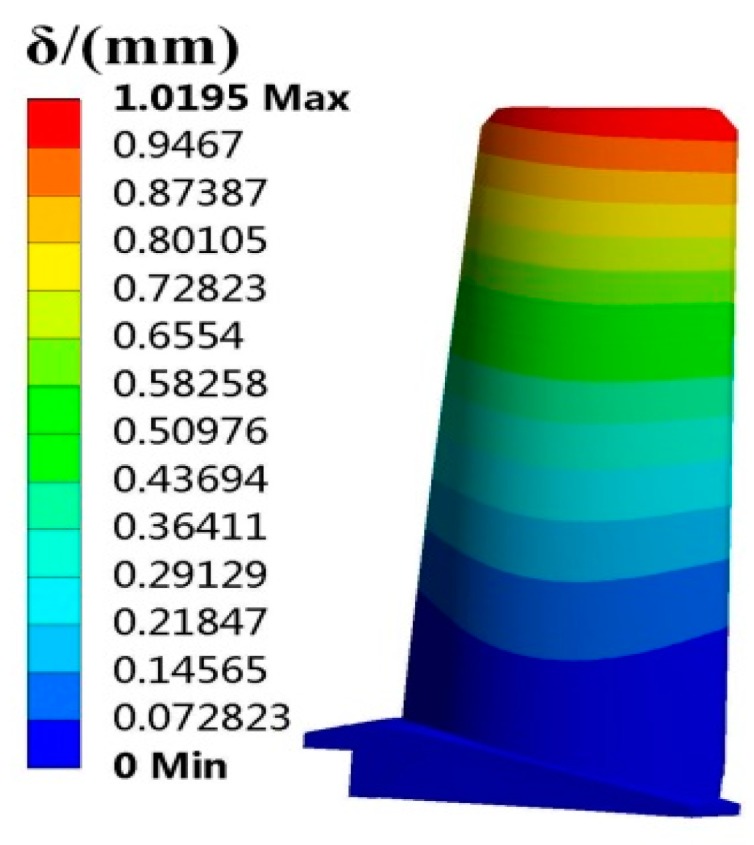
Distribution of blade deformation.

The FE model of the turbine blade was built as shown in Figure 3. The FE model includes 97,795 elements and 160,516 nodes. In term of the means of random parameters in Table 1 and the FE basic equations of the blade (shape function of tetrahedron [38], geometric equation [39], physical equation [40]), the static deterministic analyses of the blade deformation and stress were conducted, in which the deformation is radial deformation, and the stress is Von Mises stress in this paper. When the analysis reached stability, the distributions of the blade Von Mises stress and radial deformation are drawn in Figure 4 and Figure 5.

As revealed in Figure 4 and Figure 5, the maximum blade stress is 584.75 MPa at the midspan of the blade root and the maximum blade deformation is 1.0195 mm at the blade top. Therefore, the stress at the blade root and the deformation at the blade top are focused on in studying the stress and deformation failure analysis of turbine blades in blade RBDO in this paper.

### 3.2. Modeling for Fuzzy Multi-SVR Learning Method

**Figure 6:**
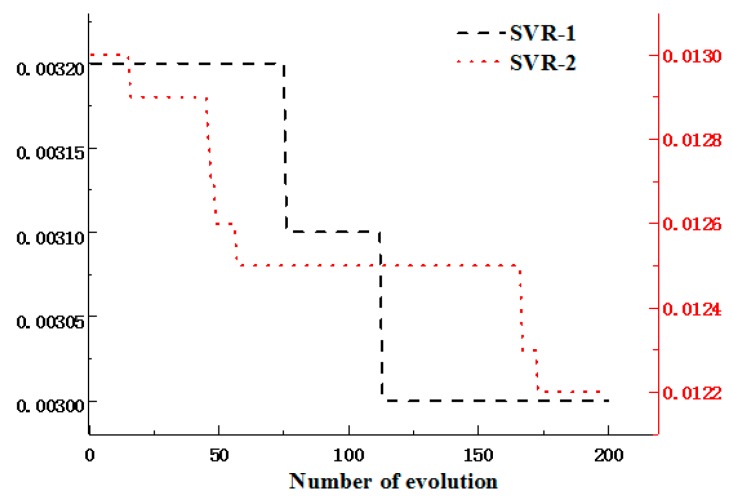
Fitness variation curves of an artificial bee colony (ABC).

The input random variables in Table 1 were sampled by LHS [36] at the critical point as the blade stress and deformation reach to the maximum. In other words, the critical points were the maximum values of the blade deformation and stress acquired by the above static deterministic analyses. The output responses of maximum stress and deformation were obtained by static analyses based on the extracted samples. The samples were normalized as the training samples for SVR modeling. To improve SVR modeling accuracy, the parameters ***θ*** = (*c*, σ, *ε*) of SVR model were optimized by an artificial bee colony (ABC) algorithm [25]. The fitness function of the ABC algorithm was defined as the mean square error (MSE) in the training process of the SVR model. Two hundred iterations were generally performed to gain the fitting curves of blade stress’s SVR (SVR-1) and deformation’s SVR (SVR-2). The two curves are drawn in Figure 6. SVR models are determined in respect of the optimal parameters (*c*, *σ*, *ε*) searched by the ABC algorithm. The coefficients of fuzzy multi-SVR learning models involving blade stress and deformation are shown in Equations (5) and (6). In coming work, we employ the two fuzzy multi-SVR learning models to carry out the RBDO of the multi-failure turbine blade.

Blade stress:

(5) $$\left\{ \begin{array}{l} a_{1}^{\left(1\right)}=\left(\begin{array}{ccc} -5.4829 & 7.9638 & -5.1672\\ -4.0834 & -4.3238 & 5.8076\\ -0.5 & 0.1041 & -5.0773 \end{array}\right)\\ \left[c_{1},\sigma_{1},\varepsilon_{1}\right]=\left[23.1011,77.3432,0.0215\right]\\ b^{\left(1\right)}=0.4527 \end{array}\right.$$

Blade deformation:

(6) $$\left\{ \begin{array}{l} a_{2}^{\left(2\right)}=\left(\begin{array}{ccc} 11.0025 & -2.8516 & 19.1313\\ 3.0836 & 6.7712 & -9.5039\\ -4.7346 & 8.5627 & 14.9729 \end{array}\right)\\ \left[c_{2},\sigma_{2},\varepsilon_{2}\right]=\left[237.7889,98.4413,0.3126\right]\\ b^{\left(2\right)}=0.1892 \end{array}\right.$$

### 3.3. RBDO of Multi-Failure Turbine Blade

**Figure 7:**
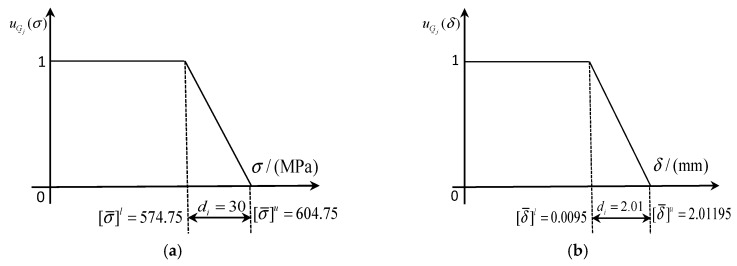
Membership functions of blade allowable failures. (**a**) Allowable stress membership function, (**b**) allowable deformation membership function.

When the allowable stress $\left[\bar{\sigma }\right]$ and allowable deformation $\left[\bar{\delta }\right]$ of blade satisfy the distribution characteristics of fuzzy membership as explained in Figure 7, the maximum stress *σ_max_* and maximum deformation *δ_max_* of the blade are minimized with regard to the RBDO model with the fuzzy multi-SVR learning method. We consider input random variables (*ω, T*) as design variables, and both blade stress *σ* and deformation *δ* as design objective function, as well as reliability index and boundary loads as constraint conditions, thus the RBDO model of turbine blade are shown in Equation (7). Herein, the upper and lower limits of fuzzy constraints are listed in Table 2.

(7) $$\begin{array}{l} \mathrm{Find}\begin{array}{cc} & \underset{˜}{x} \end{array}={\left({\underset{˜}{x}}_{1},{\underset{˜}{x}}_{2},{\underset{˜}{x}}_{3}\right)}^{T}={\left(\underset{˜}{w},\underset{˜}{T}\right)}^{T}\\ \min\begin{array}{cc} & f(x)=[{\underset{˜}{f}}_{1} \end{array}(\sigma ),{\underset{˜}{f}}_{2}(\delta ){]}^{T}\\ \text{s.t.}\;\left\{ \begin{array}{l} R_{j}\geq [R_{j0}]=[0.95,0.94]\\ R\geq [R_{0}]=[0.98]\\ \underline{w}\underset{˜}{\leq }w\leq \bar{w}\\ \underline{T}\underset{˜}{\leq }T\underset{˜}{\leq }\bar{T}\\ {\underset{˜}{f}}_{1}(\sigma )\underset{˜}{\leq }[\bar{\sigma }]\\ {\underset{˜}{f}}_{2}(\delta )\underset{˜}{\leq }[\bar{\delta }] \end{array}\right. \end{array}$$

**Figure 8:**
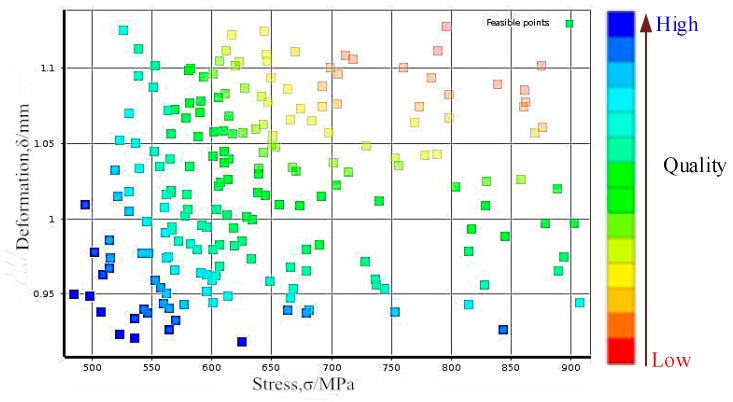
Optimal solution set of pareto.

**Figure 9:**
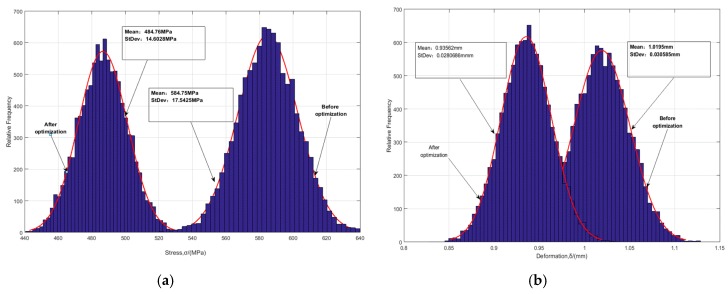
Distributions of blade stress and deformation before and after optimization. (**a**) Stress distributions, (**b**) deformation distributions.

In NSGA-II algorithm [24], we consider the coefficient of optimal front-end individual as 0.3, and the population size as 100, the evolutional generation as 200, the stop algebra as 200 and the deviation of fitness function as 10e-2. By solving the RBDO model in Equation (7), the Pareto optimal solution set of blades and the distributions of blade stress and deformation are shown in Figure 8 and Figure 9, and the optimization results are shown in Table 3. It should be noted that 10,000 simulations were conducted in each probabilistic analysis involved in the RBDO of the turbine blades with the proposed approach. 

Figure 8 indicates the quality of sample points, as the samples closing to coordinate axes are of high quality, and vice versa. In line with the Pareto picture in Figure 8, most of the sample points (optimal solutions (blue points)) set are distributed near the two axes and a spot of solutions are far from the two axes, which illustrate the high quality of the gained optimal solution set and the reliability of acquired samples. As demonstrated in Figure 9 and Table 3, under the conditions where the allowable stress and allowable deformation of the blade meet the distribution characteristics of the fuzzy membership function, the means of the optimized blade stress and deformation after optimization are obviously reduced by about 100 MPa and 0.084 mm respectively. The standard deviations of stress and deformation after optimization are weakly decreased by about 3 MPa and 0.002 mm respectively, relative to these before optimization. In this case, the optimal values of rotational speed and temperature are 1200.1 rad/s and 1110.9 K. Therefore, it is supported that the developed RBDO model with the fuzzy multi-SVR learning method is effective for the RBDO of the blade with multi-failure modes (i.e., deformation failure and stress failure). 

### 3.4. Fuzzy Multi-SVR Learning Method Validation

To verify the validity and accuracy of fuzzy multi-SVR learning method, the RBDO of overall blade failures are performed with MC simulation, traditional SVM method in [8], and the fuzzy multi-SVR learning method based on the same parameters and simulation environment. Through the comparison of the three methods, the reliability degrees and optimized objective functions are revealed in Table 4 and Table 5, respectively, under different simulations. In Table 4, the reliability degree is defined by the ratio of the number of simulations in the safety domain to the total number of simulations (1000 simulations in this paper).

As shown in Table 4 obtained from probabilistic failure analysis of the disk, (1) MC method does not have computing time when the number of simulations is larger than 10^4^, because MC method cannot perform the calculation for a too-large computational burden for probabilistic analysis of blade FE models. Thus, it is inefficient for the MC method to conduct the design analysis of complex structure with large-scale simulations, (2) time-cost for blade probabilistic analysis increases with the increase of MC simulations for three methods, (3) the time consumption of the fuzzy multi-SVR learning method is far less than those of the MC method and the SVM method for the same number of simulations. For instance, the fuzzy multi-SVR learning method only spends 0.156 s for 1000 simulations, which is only about 1/2.2 × 10^6^ that of the MC method and 47.5% that of the SVM method. Meanwhile, the strength of fuzzy multi-SVR learning method in time computation presents more obvious with increasing simulations. It is thus demonstrated that the efficiency of fuzzy multi-SVR learning method is far higher than the MC method and SVM method in calculation, and the fuzzy multi-SVR learning method is an efficient approach replacing FE models and SVM models, for the probabilistic analysis of multi-failure structures. (4) For the same simulations, the reliability degrees of blade coupling failure with the fuzzy multi-SVR learning method are almost consistent with these with the MC method, and are higher than the traditional SVM method. Moreover, the reliability degree of the blade increases and becomes higher with the rise of simulations. It is illustrated that more precise results such as reliability degree, can be gained by increasing the number of MC simulations against the response surface models, for structure design analysis from a probabilistic perspective.

As revealed in Table 5 summarized from turbine blade RBDO with stress failure and deformation failure in respect of the fuzzy multi-SVR learning method and traditional SVM method, the stress and deformation of the turbine blade are reduced by 92.38 MPa and 0.09838 mm, respectively. While the MC method reduces blade stress and deformation by 31.16 MPa and 0.03136 mm, respectively, and the traditional SVM method shortens 53.52 MP and 0.0194 mm, respectively. The comprehensive reliability index of the blade was improved from 95.4% to 96.9%, 97.83% and 98.85% for MC method, SVM method, and fuzzy multi-SVR learning method. Obviously, it is verified that the fuzzy multi-SVR learning method is workable for the fuzzy RBDO of complex structures just like the multi-failure turbine blade.

In summary, the developed fuzzy multi-SVR learning method has high modeling precision and high simulation efficiency for the comprehensive RBDO for multi-component structures. Besides, with the proposed fuzzy multi-SVR learning method, more satisfactory optimization results can be acquired for the RBDO of complex structures with multiple models, relative to MC simulation and SVM methods. Therefore, it is demonstrated that machine learning methods are promising to offer more satisfying accuracy and efficiency in modeling and RBDO of multi-failure structures or multi-model systems. 

## 4. Conclusions

The objective of this study attempts to adopt the machine learning method for the reliability-based design optimization (RBDO) of complex structure with multi-failure modes. Regarding support vector machine of regression (SVR) (a classical machine learning approach) as the basis model of RBDO, the fuzzy multi-SVR learning method was proposed by introducing fuzzy theory and the SVR model into the multi-response surface method, to effectively improve the precision and efficiency of the RBDO of multi-failure structures. We investigate the theory and modeling of the fuzzy multi-SVR learning method and give the procedure of probabilistic optimization of multi-failure structures. In respect of the fuzzy multi-SVR learning method, the fuzzy RBDO of the multi-failure turbine blade is implemented with the design objectives of blade stress and blade deformation, design variables comprising material parameters and load parameters and the fuzzy constraint boundary conditions of design objective functions. Some conclusions are summarized as follows:

(1) From the RBDO of a turbine blade with deformation and stress failures with the presented fuzzy multi-SVR learning method, we gain that the stress and deformation of the blade under operation reduced by 92.38 MPa and 0.09838 mm, in the promise of acceptable computational precision and efficiency, which is promising to improve the reliability of turbine blade.
(2) With regard to the probabilistic failure analysis of the bladed disk, we find that the developed fuzzy multi-SVR learning method does not only costs a small amount of analytical time and high computational efficiency relative to the Monte Carlo (MC) method and the SVM method, but also has an acceptable computational precision in the reliability degree as its optimization results are almost consistent with that of the FE method based on MC simulations. Moreover, the strengths of the proposed fuzzy multi-SVR learning method in modeling and simulation become more obvious with the increasing simulations.
(3) In terms of the fuzzy RBDO of the multi-failure blade, it is illustrated that the developed fuzzy multi-SVR learning method is more workable than the MC method and SVM method. The reason is that the optimal parameters including design parameters and optimization objects are preferable as larger reductions and higher reliability degree.

This study attempts to apply the machine learning method to the RBDO of multi-failure structures. Along with the heuristic thought, we develop a novel machine learning approach, fuzzy multi-SVR learning method based on fuzzy theory and SVR, and validate the promising method to be a useful way in highly-precise modeling and highly-efficient simulation by the RBDO of multi-failure blades. The efforts of this paper are promising to be conductive to enrich and develop mechanical reliability design methods and theory. 

## Figures and Tables

**Table 1:** Numerical characteristics of input random variables.

Random Variables	Mean	Standard Deviation	Distribution
Density *ρ*, kg/m^3^	8210	414.1934	Normal
Rotor speed *ω*, rad/s	1168	104.7138	Normal
Temperature *T*, K	1173.2	105.18	Normal
Aerodynamic pressure *P*, MPa	0.5	0.0448	Normal
Gravity *g*, m/s^2^	9.8	0.294	Normal

**Table 2:** Fuzzy transition constraints of design parameters.

Upper and Lower Limit		[*σ*], MPa	[*δ*], mm	*ω*, rad/s	*T*, K
Upper bound	Upper limit	604.75	2.01195	1349.0	1355.0
	Lower limit	574.75	0.00195	1284.8	1290.5
Lower bound	Upper limit	-	-	1051.2	1055.9
	Lower limit	-	-	735.84	739.13

**Table 3:** Optimization results of overall blade failure.

Design Variables	Original Data	Optimization Results
*ω*, rad/s	1168	1200.1
*T*, K	1173.2	1110.9

**Table 4:** Computing time and reliability degrees of blade probabilistic analysis with different methods.

Number of Samples	Computing Time, s			Reliability Degree, %		
	MC Method	Traditional SVM	Fuzzy Multi-SVR Learning Method	MC Method	Traditional SVM	Fuzzy Multi-SVR Learning Method
10^2^	54,000	0.0108	0.0062	99	97	98
10^3^	339,200	0.329	0.156	99.5	98.3	99.2
10^4^	-	0.789	0.468	99.34	98.65	99.29
10^5^	-	2.013	1.232	-	98.791	99.782

**Table 5:** Optimization results of multi-failure blade with different methods.

Objective Functions	Before Optimization	MC Method		Traditional SVM		Fuzzy Multi-SVR Learning Method	
		After Optimization	Reduction	After Optimization	Reduction	After Optimization	Reduction
*σ*, MPa	583.75	552.59	31.16	530.23	53.52	491.37	92.38
*δ*, mm	1.0195	0.98814	0.03136	1.0001	0.0194	0.92112	0.09838
*R*	95.40	96.9	-	97.83	-	98.85	-
